# Therapeutic Implications of Autophagy Inducers in Immunological Disorders, Infection, and Cancer

**DOI:** 10.3390/ijms18091959

**Published:** 2017-09-12

**Authors:** Sanguine Byun, Eunjung Lee, Ki Won Lee

**Affiliations:** 1 Division of Bioengineering, Incheon National University, Incheon 22012, Korea; sbyun@inu.ac.kr; 2 Traditional Alcoholic Beverage Research Team, Korea Food Research Institute, Seongnam 13539, Korea; ejlee@kfri.re.kr; 3 Advanced Institutes of Convergence Technology, Seoul National University, Suwon 16495, Korea; 4 Research Institute of Agriculture and Life Sciences, Seoul National University, Seoul 08826, Korea

**Keywords:** autophagy, immunity, cancer, activators

## Abstract

Autophagy is an essential catabolic program that forms part of the stress response and enables cells to break down their own intracellular components within lysosomes for recycling. Accumulating evidence suggests that autophagy plays vital roles in determining pathological outcomes of immune responses and tumorigenesis. Autophagy regulates innate and adaptive immunity affecting the pathologies of infectious, inflammatory, and autoimmune diseases. In cancer, autophagy appears to play distinct roles depending on the context of the malignancy by either promoting or suppressing key determinants of cancer cell survival. This review covers recent developments in the understanding of autophagy and discusses potential therapeutic interventions that may alter the outcomes of certain diseases.

## 1. Introduction

The maintenance of cellular homeostasis in response to environmental stress is crucial for the survival of all cell-based life forms. Under metabolic stresses such as nutrient or growth factor restriction, there are physiological systems in place to eliminate damaged proteins, lipids, and organelles. Many of these intra-cytosolic components can be recycled to some extent in a ‘self-eating’ process called autophagy. The process can be induced by amino acid, glucose, or oxygen starvation, growth factor deficiency, and other forms of cellular stress accompanied by cytotoxic damage, and permits the reuse of damaged proteins and organelles [1,2]. Damaged or dysfunctional intracellular components including unfolded proteins are ubiquitinated for selective recognition by autophagy receptors [3]. Upon recognition, the damaged components are then exported to lysosomes for autophagic degradation and delivery to the cytoplasm where they are recycled. As a self-cleansing mechanism, autophagy is indispensable for ensuring the integrity of proteins and organelles, as well as energy homeostasis [2,4].

Autophagy is categorized into three major types. Microautophagy is a non-selective degradation process that involves the direct engulfment of small volumes by lysosomes. Chaperone-mediated autophagy (CMA) is a selective degradation process that recognizes and translocates specific proteins to the lysosome through lysosomal-associated membrane protein 2 (LAMP2), a receptor for the CMA pathway. Macroautophagy involves double-membrane bound autophagosomes and targets damaged components in the cytoplasm for degradation. This is the most well-characterized form of autophagy and the type primarily discussed in this review.

In normal tissues, low levels of basal autophagy catabolize damaged cellular components to maintain balanced cellular homeostasis. Multiple studies have linked autophagy and the pathology of various diseases including cancer, neurodegenerative diseases, infectious disease, autoimmunity, and aging [5,6]. Here, we briefly review the pivotal roles of autophagy in regulating immunity and cancer, and discuss the potential for autophagy inducers to be developed as therapeutic agents.

## 2. Role of Autophagy in Immunity

Autophagy as a fundamental process for maintaining cellular integrity and is intimately linked with immune responses. The deletion of autophagy signaling factors such as the autophagy-related gene (*Atg*) leads to defects in immune responses and related pathogenesis. T cell-specific deletion of *beclin 1* (*Atg6*) in mice fails to induce T cell-dependent humoral immune responses and resistance to encephalomyelitis [7]. Mice lacking *Atg16L1* in haematopoietic cells are prone to acute colitis induced by dextran sodium sulphate through endotoxin-induced inflammasone and IL-1β secretion, highlighting the crucial role of autophagy in endotoxin-induced inflammatory immune responses [8]. There have been multiple proposed mechanisms by which the immune system regulates autophagy, including the direct removal of microbes during infection, antigen presentation, lymphocyte homeostasis, and the control of inflammation [9,10].

Recognition of infected microbes by pathogen-recognition receptors such as TLRs, or NLRs (NOD-like receptor) induces autophagy to facilitate their elimination [6]. Activation of TLR4 or TLR7 by LPS or single-stranded RNA (ssRNA) leads to the formation of autophagosomes and eliminates mycobacteria, showing a strong association between autophagy and innate immunity via TLR signaling [11]. LPS-stimulated TLR4 triggers the activation of MyD88- and TIR domain-containing adapter-inducing interferon-β (TRIF)-dependent signaling which in turn mediates upregulation of nuclear factor kappa B (NF-κB) and activator protein 1 (AP1) signaling, leading to innate immune defenses such as pro-inflammatory cytokine production [12]. NLRP4, a member of the NLR family, detects bacterial infection and induces Beclin 1-mediated autophagy in response [13]. Recently, the role of autophagy in the expression of resistance to *Mycobacterium tuberculosis* was reported [14,15]. Mice with macrophage-specific deletion of *Atg5* were more susceptible to infection against *M. tuberculosis* and developed severe inflammation in the lungs [16]. However, other autophagy signaling genes including *Atg14*, *Atg12*, *Atg16L1*, *Atg7*, and *Atg3* had little effect on survival during *M. tuberculosis* infection, suggesting the possibility of a specific role for *Atg5* against neutrophil-mediated inflammation in an autophagy-independent manner. These findings also imply that autophagy genes might not all play critical roles during mycobacterium infection [17].

In the adaptive immune response, autophagy also plays an essential role in antigen processing and presentation through major histocompatibility complexes (MHC). Traditionally, it is known that MHC-I presents antigens derived from intracellular proteasomal proteins to CD8^+^ T cells, whereas MHC-II displays extracellular antigens and presents to CD4^+^ T cells for lysosomal degradation [18]. Recently, the emerging role of autophagy for presentation of intracellular antigens by MHC-II has been reported [19,20]. Approximately 10–25% of MHC-II presents cytoplasmic and nuclear antigens via autophagy, and several mechanisms are proposed by previous studies [20,21]. Cytoplasmic antigens are degraded by cytosolic proteases and processed by factors in the CMA pathway including LAMP-2A and hsc70 to access lysosomes [22]. Via macroautophagy, intracellular antigens are sequestered and encapsulated by autophagosomes that bind to Atg8/LC3, which then interacts with MHC-II to form MHC-II compartments (MIIC) for lysosomal degradation [21]. MHC-II molecules are combined with invariant chains (Ii) in the endoplasmic reticulum (ER), and transported to MIIC by fusing with late endosomes and autophagosome-lysosomes. The antigen fragments captured by MHC-II are catalyzed by the MHC-encoded molecule HLA-DM in MIIC, which delivery of peptide fragment-MHC-II complexes to the cell surface for surveillance by CD4^+^ T cells [21,23].

Autophagy can also regulate the homeostasis of lymphocytes. More specifically, it is involved in the development, activation, survival, and differentiation of T lymphocytes [24]. The maintenance of organelle homeostasis in T cells is one of the crucial roles of macroautophagy. The inhibition of macroautophagy by deletion of *Atg7* in T cells induces defective survival, enhances mitochondrial contents, increases ROS production, and dysregulates the expression of apoptotic proteins [25,26]. *Atg7*-deficiency in T cells also induces the accumulation of content in the ER and calcium stores, showing the importance of autophagy in maintaining ER and calcium homeostasis in T lymphocytes [27].

Autophagy plays an important role for T cell survival. Loss of *Atg5* in T cells leads to the accumulation of abnormal proteins and organelles in the cytoplasm and stimulates cell death via the generation of ROS and impaired mitophagy [26,28]. Disruption of Beclin 1 in T cells induces an increased accumulation of the pro-apoptotic proteins Bim, caspase-3 and -8, further support that under the absence of autophagy pathways, abnormal survival and apoptotic responses occur in T cells [29].

The full activation of T cells requires several signaling steps including the engagement of antigens with T cell receptors (TCR) via MHC, and subsequent engagement of co-stimulatory molecules including CD28 and IT2 signaling. Previous studies have demonstrated the importance of TCR engagement on CD4^+^ and CD8^+^ T cells for the induction of macroautophagy [30,31]. Full activation of T cells by TCR engagement upregulates the lipidated form of LC3 (LC3-II) in an mechanistic target of rapamycin (mTOR)-independent manner [24,30]. Selective degradation of specific proteins by p62-mediated macroautophagy is caused by modulation of Bcl10, which is downstream of the TCR. TCR engagement promotes ubiquitination of Bcl10 and binding with p62, which gradually induces degradation by autophagy. Silencing of p62 blocks Bcl10 degradation, and disruption of Atg3 in CD4^+^ T cells reduces TCR-mediated Bcl10 degradation, enhancing NF-κB activation [32]. In vivo studies using mice models with a deleted *Atg7* gene in T cells revealed defective production of IL-2 and IFN-γ and decreased proliferation in response to T cell stimulation [31]. In contrast, there are several reports showing an opposite role for TCR activation in autophagy. T cell activation by TCR engagement can induce proliferation with very low levels of autophagy activity [33]. In addition, Bcl10-mediated autophagy regulates TCR activation of NF-κB [32,34], further demonstrating the complexity of TCR activation in autophagy modulation.

An emerging role for autophagy in the formation of memory CD8^+^ T cells has also been suggested in recent studies. The loss of *Atg5* and *Atg7* in mice triggers the formation of memory T cells with defects in survival during the contraction phase to memory phase transition, suggesting that autophagy is an essential process for antigen-specific effector CD8^+^ T cell memory formation [33]. Another study reported that autophagy in CD8^+^ T cells is dramatically reduced in aged mice, and deficiency in the *Atg7* autophagy gene leads to a failure of memory formation in CD8^+^ T cells against influenza and MCMV infection [35]. In order to examine the mechanism responsible, metabolic and transcriptomic analyses were performed and it was shown that defects in autophagy (Atg7-deficient CD8^+^ T cells) caused dysregulation in lipid biosynthetic pathways in T cells during transition to the memory phase. Furthermore, this alteration in metabolic homeostasis may hinder growth factor signals required for memory T cell development [33]. In addition, Atg7^−/−^ antigen-specific CD8^+^ T cells displayed problems in regulating metabolic markers and maintaining healthy mitochondria compared to their wild-type counterparts [35].

Autophagy also functions to control the balance of abnormal immune activation and inflammation [6]. Epidemiological studies in Caucasian and Chinese populations demonstrated a positive association between Atg5 expression and systemic lupus erythematosus (SLE) by meta-analysis [36]. Activation of the autophagy signaling proteins Beclin 1 and Atg7 has been reported in the pathogenesis of rheumatoid arthritis [37]. By genome-wide association studies, *ATG16L1*, *NOD2*, and *IRGM* were identified as major risk factors of inflammatory Crohn’s disease (CD) [38]. The variation (T300A) in *ATG16L1* causes an autophagy-associated defect in the function of Paneth cells, which could be potentially linked with the pathogenesis of CD [39]. Loss of Atg16L1 in mice led to increased production of pro-inflammatory cytokines IL-1β and IL-18 by macrophages [8]. These studies indicate that autophagy might be crucial in the control of immunity and inflammation. Although further mechanistic studies for autophagy-regulated immune-related molecules are needed, evidence suggests that beclin 1 might be disrupted by TLR adaptors, and NOD1/2 interacts with Atg16L1 and recruits LC3 [6,40].

As immunosurveillance plays a crucial role in carcinogenesis, the control of immune responses by autophagy can be a critical factor in determining the fate of cancer cells [9,41]. Tumors emerge by evading immune surveillance and altering their antigenic properties, as well as by suppressing anticancer immunity [42]. Recently, the activation of host immunity to treat cancer has been recognized as one of the most promising novel therapeutic approaches [43]. Because autophagy is deeply involved in the proper functioning of various immune cells, controlling the autophagy pathway may stimulate immune reactions against tumor cells [9]. Also, malignant cells with autophagy deficiencies have been reported to suppress the recruitment of immune cells after chemotherapy, reducing anticancer immunogenicity [44].

Taken together, these reports suggest that autophagy as a central modulator of immunity could be harnessed for the treatment of various immune-mediated inflammatory disease including bacterial and viral infections, transplantation rejection, cancer immunotherapy, and other inflammatory disorders.

## 3. Role of Autophagy in Cancer

Autophagy is clearly indispensable for homeostasis and survival in times of cellular stress in normal tissues. However, in some situations, it also enables cancer cells to circumvent programmed cell death [45]. Due to their unrestrained cell growth, cancer cells typically exhibit high energy demands which can be supported through alternative metabolic pathways, including autophagy. With defects in apoptosis, cancer cells can tolerate long-term metabolic stresses including glucose, cytokine, and oxygen deprivation, and can overcome such restrictions by relying on nutrient recycling mechanisms mediated by autophagy. Detecting environmental nutrient status is crucial for autophagy, and mTOR and AMP-activated protein kinase (AMPK) signaling act as key nutrient sensors in the autophagic machinery [46]. Nutrient and growth factor-induced mTOR complex 1 (mTORC1) activation inhibits autophagy by phosphorylating relevant target proteins such as unc-51-like kinase 1 (ULK1), ATG13, and ATG14L, thereby promoting cell growth [47,48]. Conversely, autophagy is induced by AMPK through the direct phosphorylation and activation of ULK1 on Ser 317 and Ser 777 under nutrient deprivation, whereas the hyperactivation of mTOR suppresses ULK1 by inducing phosphorylation at Ser 757 [49].

Autophagy has been reported to play dual roles in cancer, showing both anti-tumor and tumor-promoting effects depending on the carcinogenic stage and tissue involved [50]. Loss-of-function in the autophagy pathway via genetic mutation or knockdown of autophagy regulators such as beclin 1, Atg5, and Atg7, or constitutive activation of PI3K or mTOR signaling promotes cell survival and the proliferation of adenomas and carcinomas [1,51,52,53]. Liver-specific deficiency of *Atg7* and mosaic deletion of *Atg5* in mice causes the spontaneous development of liver adenomas marked by p62 accumulation, the induction of oxidative stress, and genomic instability [54]. *Beclin 1* is also a key mediator of autophagy, and mutations in this gene can be found in 40–75% of human sporadic mammary, ovarian, liver, and prostate cancers [55,56,57]. The heterozygous loss of *Beclin1* in mice causes the spontaneous development of lymphomas, colorectal carcinogenesis, and hepatitis B virus-induced hepatocellular carcinoma [56,58,59]. However, genetic deletion of single autophagy proteins such as *Atg5* or *Atg7* only induces the formation of benign liver tumors without additional tumors in other organs, and cells isolated from *Beclin1*^+/−^ mice still exhibited autophagic activity [54,56]. Thus, it is difficult to determine the role of autophagy in tumor suppression based on observations from models of genetic deletion of a single autophagy protein.

Multiple reports have suggested that autophagy can be used to evade cell death in some cancer cells to survive during environments with limited energy. Various cancer cells have high levels of basal autophagy even under nutrient-rich conditions, highlighting a more complex role for autophagy in regulating cancer cell survival. Autophagosome formation is known to be critical for the survival of some tumors, especially in hypoxic regions [60,61], playing a major role in long-term tumor survival and re-growth after chemotherapy by allowing residual tumor cells to recover or metastasize. Crosstalk exists between the autophagy and apoptosis pathways, and cancer cells with defects in the apoptosis pathway can feature upregulated autophagy dependence [62]. In apoptosis-defective cancer cells, blocking autophagy can trigger acute necrotic cell death arising from a failure to withstand metabolic stress, indicating that autophagy may represent a therapeutic target for selective cancer treatment [62,63,64]. Selective tumor treatment is possible by combining autophagy inhibitors with metabolic stress to sensitize tumor cells to cancer-specific necrotic cell death [63]. The inhibition of angiogenesis, growth factors, and related receptors common in many current cancer therapies often promotes intense metabolic stress on the glycolysis pathway [1]. Metastatic tumor cells have high energy demands to enable proliferation, migration, invasion, and angiogenesis, and residual cancer cells remaining after chemotherapy or the surgical removal of tumors also require favorable conditions for re-growth and survival during times of energy restrictions. In mouse models, the suppression of autophagy by chloroquine (CQ; a lysosome acidification inhibitor) promotes p53 activity by inducing apoptotic cell death in lymphoma cells [65]. Combination therapies involving anti-cancer agents such as tamoxifen, bortezomib, 5-FU, cisplatin, imatinib, rapamycin, and hormone therapy with autophagy inhibitors like 3-methyladenine (3-MA), BAF (bafilomycin A), CQ, or the knockdown of autophagy signaling mediators (*ATG5*, *ATG6*, and *ATG7*) by siRNA significantly inhibits cancer cell growth and metastasis in breast, colorectal, prostate, skin, and gastric cancers [66,67,68,69]. These results strongly suggest that inhibition of autophagy signaling may form part of a promising combination therapy for malignant, metastatic or residual cancer cells.

Despite the beneficial effects of autophagy inhibition toward sensitizing cancer cells, autophagy still acts as a barrier against the generation of abnormal proteins and organelles in normal cells. Precisely how autophagy upregulation suppresses tumorigenesis is only partly understood, but several potential mechanisms have been proposed. Loss-of-function in autophagy signaling mediators such as *Beclin 1*, *Atg5*, and *Atg7* is strongly associated with tumor initiation and progression [57,70]. Conversely, over-stimulation of autophagy in tumors leads to excessive cellular damage and triggers autophagic cell death [71]. There are numerous potential benefits for autophagy activation in tumorigenesis. Autophagy limits genome instability and mutations by inducing apoptosis-independent cell death and eliminating damaged intracellular components. Under starvation conditions, autophagic cell death and the recycling of components is crucial for DNA damage repair and the maintenance of cellular homeostasis. Defects in endoplasmic reticulum (ER) chaperones and p62 accumulation in tumor cells induces reactive oxygen species (ROS) production and genome damage [71]. To eliminate damaged mitochondria, a specific form of autophagy in mitochondria, known as mitophagy, is activated by the PTEN/PINK1/PARK2 signaling pathway, and involves the ubiquitylation of damaged mitochondrial outer membrane proteins [72]. Moreover, as a crucial activator of nuclear factor erytheroid-derived-2-like 2 (NRF2), p62 interacts with Keap1-NRF2 signaling. Liver-specific loss of autophagy function through *Atg7* deletion induces the nuclear accumulation of NRF2, which stimulates NRF2-related gene expression and promotes tumorigenesis by p62 accumulation [54,73,74]. Defects in autophagy that promote oxidative stress and chronic tissue damage also lead to an upregulation of inflammatory cytokines and the promotion of tumor cell growth [75,76]. Abnormal increases in inflammation are associated with cancer progression under autophagy-restricted conditions, suggesting that combination therapies with anti-inflammatory agents and autophagy activators for cancer treatment might suppress cancer growth with a combination of calorie restrictive conditions and exercise [2]. Depending on the context, such as tumor type and stage, it appears that autophagy can have varying effects on cancer development. However, it is evident that autophagy plays a major role in cancer cell survival and therefore the development of autophagy-targeting agents is likely to be therapeutically useful if applied in the right context.

## 4. Activators of Autophagy for Immunity

### 4.1. BRD5631

Using a high-throughput screening system based on the assessment of GFP-LC3 punctae per cell, BRD5631 and other compounds with autophagy-activating activity have been identified [77]. The hit compounds identified promoted autophagy without inhibiting the mTOR signaling pathway. Autophagy is known to enhance the clearance of bacterial pathogens [9] and BRD5631 and two other selected compounds were observed to promote antibacterial autophagy. These compounds were also able to inhibit replication of *Salmonella* without showing direct bactericidal activity, and suppressed IL-1β secretion induced by lipopolysaccharide and muramyl dipeptide in macrophages. As these compounds work independently of the mTOR pathway, using these autophagy modulators may facilitate the regulation of immune responses in situations where inhibiting mTOR would cause undesirable side effects.

### 4.2. Spermidine

Spermidine is a natural polyamine found in various foods including soybean, broccoli, cauliflower, chicken, steak and potato [78]. Spermidine promotes cardioprotection and lifespan extension in yeast, flies, worms, and mice [79,80]. The primary mechanism responsible for these health-promoting effects is suggested to be the autophagy-enhancing ability of spermidine in an mTOR-independent manner [79,80,81]. In addition, spermidine has also been implicated in the control of immune responses. Mice lacking the autophagy gene *Atg7* in T cells exhibit defective memory CD8^+^ T cell formation in response to influenza and murine cytomegalovirus infection [35]. The study also showed that old mice had reduced memory CD8^+^ T formation and decreased autophagic flux. However, treatment with spermidine induced autophagy in T cells and increased influenza-specific CD8^+^ T cell responses in old vaccinated wild-type mice, but not in the old vaccinated T cell-*Atg7* knockout mice. While mTOR inhibition by rapamycin has been reported to stimulate the T cell memory response as well [82], spermidine was shown to activate autophagy and improve memory T cell generation without affecting the mTOR pathway. These results demonstrate that spermidine may be used to improve immunity through the activation of autophagy, especially in the aged population [35].

### 4.3. Trehalose

Trehalose is a disaccharide found in microorganisms, insects, invertebrates, and plants, and has been reported to induce autophagy via an mTOR-independent pathway [83]. Human cytomegalovirus (HCMV) can infect various types of cells and is a major cause of viral birth defects [84]. Additionally, HCMV has been linked with the development of atherosclerosis and brain tumors [85,86,87]. A previous study has shown that activating autophagy by trehalose may represent a potential therapeutic approach for treating HCMV disease [88]. Trehalose increases the number of autophagosomes and autolysosomes in HCMV-infected cells and suppresses HCMV replication and viral spread. It was also demonstrated that autophagy-inducing activity as well as inhibitory effects against HCMV were reproducible in three different cell types. Upregulating the host autophagy pathway as an antiviral approach using a natural compound is a promising approach, but autophagy inducers do not always provide benefit against viral infections. For example, trehalose-mediated autophagy was reported to impair the anti-viral response against human rhinovirus (HRV). HRV is a major cause of acute exacerbations of respiratory diseases, including asthma, chronic obstructive pulmonary diseases, and cystic fibrosis [89,90]. In the case of HRV infection, trehalose-induced autophagy led to the inhibition of anti-viral IFN-λ1 expression and promotion of HRV-16 replication in normal human tracheobronchial epithelial cells [91]. As observed in the case of trehalose, autophagy can function as both a pro-viral and an anti-viral depending on the virus and context [92]. Hence, activators of autophagy could be considered as therapeutic options against viral infections in a selective manner.

### 4.4. Resveratrol

The modulation of autophagy by resveratrol has been reported in various settings and studies have focused on its effects on immune function. A study involving a restrained mouse model with an induced splenic immunocompromised state examined the protective effect of resveratrol [93]. Restraint-stressed mice exhibited splenic damage, reduced CD4^+^ T-cell counts, and a decline in the number of splenocytes. Resveratrol administration reversed both the spleen index and overall splenocyte numbers induced by restraint and increased the number of CD4^+^ T cells. It was further confirmed that resveratrol induced autophagy in the mouse spleen. In another study, resveratrol was reported to suppress vascular endothelial inflammatory reactions [94]. Resveratrol prevented tumor necrosis factor α-induced inflammation by inducing autophagy in human umbilical vein endothelial cells. The study used siRNA knockdown to investigate the molecular mechanisms responsible and found that resveratrol activates the cAMP-PRKA-AMPK-SIRT1 signaling pathway to control autophagy.

### 4.5. Vitamin D3

The active form of vitamin D, 1α,25-dihydroxyvitamin D3 (1α,25-(OH)2D3), is a pleiotropic hormone that controls multiple functions in the body and has been found to be useful for the treatment of various diseases including rickets, osteoporosis, psoriasis, cancers, cardiovascular diseases, autoimmune diseases, and infectious diseases [95]. Tuberculosis is caused by the bacteria *Mycobacterium tuberculosis*, and is one of the top 10 causes of preventable death worldwide (the World Health Organization estimates that 1.8 million died from the disease in 2015). Progression of the disease is largely determined by the host immune response [96]. A previous study demonstrated that the induction of autophagy may contribute to 1α,25-(OH)2D3-mediated antimycobacterial immunity [97]. 1α,25-(OH)2D3 activates autophagy in monocytes and macrophages, and removal of *Mycobacterium tuberculosis* by 1α,25-(OH)2D3 in monocyte-derived macrophages and the THP-1 cell line was dependent on autophagy activation. Human cathelicidin was found to play a key role in 1α,25-(OH)2D3-induced autophagy by regulating the expression of autophagy-related genes such as *Beclin-1* and *Atg5*. Human cathelicidin was also required for 1α,25-(OH)2D3-induced colocalization of mycobacterial phagosomes with autophagosomes in monocyte-derived macrophages. These findings suggest that vitamin D triggers autophagy to activate innate immunity against infection by intracellular *Mycobacterium tuberculosis*.

## 5. Activators of Autophagy for Cancer Therapy

### 5.1. Rapamycin and Rapalogs

Rapamycin is a naturally-occurring mTOR inhibitor, and its analogs (rapalogs) everolimus (RAD-001), temsirolimus (CCI-779), and deforolimus (AP-23573) inhibit mTORC1 to stimulate autophagy [46]. Everolimus was reported to suppress proliferation and enhance the anti-proliferative effect of paclitaxel when combined for the treatment of endometrial cancer cells. Co-treatment with the autophagy inhibitor chloroquine or transfection with shRNA targeting Atg5 reduced everolimus-mediated cell death, suggesting that everolimus induces autophagic cell death in endometrial cancer cells [98]. In another study, rapamycin was shown to sensitize A549 lung cancer cells to radiation therapy by inducing autophagy and delaying DNA damage repair [98]. Rapamycin activated autophagy, but not apoptosis in malignant glioma cells. Combination treatment with PI3K/Akt inhibitors synergistically enhanced rapamycin-sensitive and -resistant glioma cells to rapamycin by promoting autophagy [99]. Although it is well known that attenuating the mTOR pathway triggers autophagy, since mTOR controls various other cellular pathways including proliferation, protein synthesis, metabolism, and lipid synthesis [100], whether the induction of autophagy is solely responsible for any observed anti-cancer effects by rapalogs should be interpreted with caution. For example, everolimus-mediated autophagy was reported to contribute to a reduction in the anti-proliferative effect caused by everolimus against aromatase inhibitor-resistant breast cancer cells. Additionally, simultaneous treatment of an autophagy inhibitor with everolimus improved the efficacy of everolimus treatment against breast cancer cell proliferation [101]. In the clinic, some partial responses have been seen with rapalog treatment in renal cell carcinoma and mantle-cell lymphoma [102,103]. However, in many cases, single treatment with rapalogs has shown only modest clinical outcomes in various cancer types [46,104,105,106,107]. Collectively, rapalogs represent potent inducers of autophagy via suppression of the mTOR pathway, however, their clinical application as autophagy activators requires further investigation.

### 5.2. Metformin

*Galega officinalis* (French lilac) contains high concentrations of guanidine, and has been used as a herbal treatment for the symptoms of diabetes in Europe for centuries [108]. Metformin was first synthesized as a derivative of guanidine. The biguanide metformin lowers glucose levels and improves insulin sensitivity, and is the most frequently prescribed antidiabetic drug in the world [109]. In addition to its usage as a medicine for diabetes mellitus, metformin has gained attention as a potential agent for cancer therapy [109]. Among the suggested mechanisms of action, metformin has been implicated to suppress several types of cancers at least in part by inducing autophagy. Metformin was reported to reduce the proliferation of melanoma cells and induce autophagy [110]. While short term treatment with metformin caused cell cycle arrest at G0/G1 phase, 96 h of metformin treatment induced apoptosis. Attenuation of autophagy through silencing *LC3* or *ATG5* led to a reduction in metformin-mediated anti-proliferation and apoptosis in melanoma cells. The administration of metformin inhibited melanoma tumor growth in mice and increased autophagy and apoptosis markers in tumor samples. Similar effects for metformin in an endometrial cancer cell line were observed [111]. Metformin induced cell cycle arrest and decreased cell viability in Ishikawa endometrial cancer cells, triggering autophagy. Knockdown of Beclin-1 or co-treatment with a pharmacological inhibitor of autophagy partially reversed the cytotoxic effects of metformin. In another study, metformin was reported to induce autophagic flux and enhanced TNF-related apoptosis-inducing ligand (TRAIL)-mediated apoptosis in lung adenocarcinoma cells [112]. Either treatment with an autophagy inhibitor or silencing of ATG5 partially reversed the sensitizing effect of metformin on TRAIL-induced tumor cell death. The primary molecular mechanism responsible for inducing autophagy is thought to be intimately associated with activation of the AMP-activated protein kinase (AMPK). Activated AMPK switches the cell from an anabolic to a catabolic state, and triggers autophagy by regulating mTORC1, ULK1, and p53 signaling pathways [113,114,115].

### 5.3. Obatoclax (GX15-070)

Obatoclax is a small-molecule indole bipyrrole compound that targets Bcl-2 protein family members [116]. Obatoclax has been shown to exhibit anti-cancer activity against hematologic malignancies and overcomes glucocorticoid resistance in acute lymphoblastic leukemia [116,117,118]. The induction of autophagy was shown to be a primary mechanism of cell death elicited by obatoclax [118,119,120,121]. Obatoclax induces necroptosis by stimulating the assembly of necrosomes in autophagosomal membranes [119]. Preventing autophagosome formation by knockdown of *Atg5* or *Atg7* blocked obatoclax-mediated cell death. It was found that obatoclax triggers the interaction of Atg5 with components of the necrosome including Fas-associated death domain (FADD), receptor-interacting protein 1(RIP1) and RIP3. Further examination showed that obatoclax-induced autophagic cell death through RIP1 and RIP3 [119]. Another study demonstrated that silencing of beclin-1 or treatment with an autophagy inhibitor blocked obatoclax-mediated cell death in B-cell lymphomas, further highlighting the importance of autophagy activation in the anti-cancer effects of obatoclax [122].

### 5.4. Liensinine, Isoliensinine, Dauricine and Cepharanthine

A number of natural alkaloid compounds have been discovered to induce autophagy in cancer cells. Liensinine, isoliensinine, dauricine, and cepharanthine promote autophagic activity by increasing autophagic flux and autophagosome formation [123]. The four alkaloids were shown to induce autophagy in various types of cancer and normal cells, showing a range of IC50 values against different cell types. Treatment with the alkaloids led to an increase in AMPK phosphorylation and subsequent downregulation of the mTOR pathway, suggesting that the compounds target the AMPK-mTOR pathway to promote autophagy. The study also used several cells with an apoptosis-resistant phenotype (e.g., caspase-3, -7, -8 deficient MEFs and Bax-Bak double knockout MEFs) to examine the cytotoxic effect of the alkaloids. Isoliensinine, dauricine, and cepharanthine induced cell death in apoptosis-defective or apoptosis-resistant MEFs.

### 5.5. Maprotiline and Fuoxetine

Antidepressants have been proposed to elicit various anti-cancer effects [124,125,126,127]. A previous study reported that the antidepressants maprotiline and fluoxetine inhibit the growth of Burkitt’s lymphoma cells by inducing autophagic-Type II programmed cell death [128]. The study found that the antidepressants promoted autophagic cell death in some Burkitt′s lymphoma cells, while causing apoptosis in another Burkitt′s lymphoma cell line, suggesting that the mechanism is cell-type specific. The autophagic cell death observed in the cell line tested required an increase in extracellular calcium influx. These results suggest that cancer cells that are apoptosis-resistant can be targeted by agents that induce autophagic cell death such as maprotiline and fluoxetine.

## 6. Conclusions

Autophagy is a homeostatic degradation process that captures, degrades, and recycles cellular proteins and organelles. Autophagy has dual functions in cancer cells, whereby its activation can promote survival by reducing the impact of various cellular and environmental stresses, and in other circumstances can cause cancer cell death. It can also protect against infections, control autoimmune diseases, and regulate inflammatory responses. Therefore, activators of autophagy may potentially contribute to the control of immunity and cancer (Table 1). However, with a plethora of complex pathways influenced by autophagy, the exact mechanisms of action and the outcomes involved must first be clearly elucidated. Future studies need to focus on dissecting the detailed circumstances of when activators and inhibitors of autophagy may elicit desirable therapeutic responses.

## Figures and Tables

**Table 1:** Activators of autophagy and their mechanism of action.

Compound	Mechanisms of Action	Model (Related Disease)	References
Apigenin	AMPK activation, mTOR inhibition	Human keratinocytes	[129]
	Downregulation of TNF-α, IL-6 and IL-1β secretion	ApoE^−/−^ mice (atherosclerosis)	[130]
	Beclin-1 accumulation, conversion of LC3 protein, p62 degradation, enhanced ROS production	BCPAP cell (human papillary thyroid carcinoma)	[131]
	mTOR inhibition	Human keratinocytes (UV-induced skin cancer)	[132]
Arsenic trioxide	BNIP3 upregulation	U373-MG, U87-MG, and T98Gcell (malignant glioma)	[133]
BRD5631	IL-1β suppression, mTOR-independent autophagy activation	HeLa cell expressing LC3, hiPSC-derived neurons	[77]
Cepharanthine	AMPK activation	MEFs and cancer cells	[123]
Curcumin	Akt/mTOR inhibition	U87-MG and U373-MG (malignant gliomas)	[134]
	Stimulation of LC3-II, down-regulation of p62	Primary human umbilical vein endothelial cells, rat model of carotid artery intimal injury	[135]
	Upregulation of the JNK signaling pathway	MG63 cell (human osteosarcoma)	[136]
	Increased Atg8	Sf9 (Spodoptera frugiperda) insect cell (pest control)	[137]
	Suppression of AKT/mTOR/p70S6K signaling	A375 and C8161 cells, A375-cell transplanted mice (human melanoma)	[138]
	Iron deprivation-dependent autophagy induction	DU145 and PC3 cells (castration-resistant-human prostate cancer)	[139]
Dauricine	AMPK activation	MEFs and cancer cells	[123]
Delphinidin	Atg5-dependent accumulation of LC3-II	Atg5-deficient and normal mouse embryonic fibroblasts	[140]
Fisetin	LC3-II accumulation (synergistic effect with paclitaxel)	A549 cells (lung cancer)	[141]
	mTOR inhibition	PC3 cells (human prostate cancer), TU212 (laryngeal carcinoma)	[142,143]
Galangin	Inhibition of PI3K/Akt/mTOR signaling	TU212 and M4e cells (human laryngeal cancer)	[144]
Genistein	Activation of LKB1/AMPK pathway, inhibition of mTOR signaling	Human vascular smooth muscle cells (atherosclerosis)	[145]
Gossypol	Accumulation of Beclin 1	Human pharynx, tongue, and salivary gland cancer cell lines (Head and neck carcinoma), SALTO cells and Balb/c mice transplanted SALTO cells (mouse salivary gland cancer)	[146]
Grandifloracin	Akt/mTOR inhibition	PANC-1 cell (human pancreatic cancer)	[147]
Guttiferone K	Akt/mTOR inhibition	HeLa cell (human cervical cancer)	[148]
Isoliensinine	AMPK activation	MEFs and cancer cells	[123]
Kaempferol	Accumulation of LC3-II, ROS-mediated JNK activation	SH-SY5Y and primary neurons cells (Parkinson’s disease), HeLa (human cervical cancer)	[149,150]
	AMPK activation, mTOR inhibition	SK-HEP-1 cell (human hepatic cancer), palmitic acid-stressed RIN-5F cells and murine pancreatic islets (type 2 diabetes)	[151,152]
Liensinine	AMPK activation	MEFs and cancer cells	[123]
Magnolol	PI3K/PTEN/Akt inhibition	H460 cells (human lung cancer)	[153]
Maprotiline and Fuoxetine	Calcium influx-mediated autophagy	DG-75 cells (human Burkitt’s lymphoma)	[124,128]
Metformin	Downregulation of Src/CEBPD pathway, AMPK activation	Huh-7 cells (hepatocellular carcinoma)	[154]
	AMPK activation, inhibition of pro-inflammatory pathway (NLRP3, TNF-α, IL-6)	SH-SY5Y cells, probenecid-induced mice model (Parkinson’s disease)	[155]
	downregulation of c-FLIP and decrease in p62	A549 cell (human lung adenocarcinoma)	[112]
Obatoclax	Activation of Bax and Bak	Phase I clinical study (lymphocytic leukemia), ituximab/chemotherapy-sensitive, -resistant cell lines, and primary tumor-cells derived from patients (human lymphoma)	[116,122]
Oridonin	GLUT1-mediated and AMPK-independent autophagy induction	HCT-15, COLO205, HCT116, RKO, SW480, and SW620 cell line (Colorectal cancer)	[156]
Pentagalloylglucose	mTOR inhibition	Atg7^−/−^ MEF and knock out mice (HSV-1 infection), PC3 and TRAMP-C2 (prostate cancer)	[157,158]
Pterostilbene	JNK1/2 activation, inhibition of Akt, ERK1/2, and p38	SAS and OECM-1 cells (human oral cancer)	[159]
	accumulation of LC3-positive vacuolar structures	HL60 cell (human leukemia)	[160]
Quercetin	H-RAS degradation	Ha-RAS-transformed Caco-2 cell (colon cancer)	[161]
	Reduced p62 protein expression	A549 cell (lung cancer)	[162]
	Ameliorated ER stress and oxidative stress	Rotenone-induced rat model (Parkinson’s disease)	[163]
	Inhibition of PI3K/Akt/mTOR and STAT3 signaling	PEL cell (primary effusion lymphoma)	[164]
Rapamycin	increased Beclin-1 and PINK1	Mild traumatic brain injured rat model	[165]
	Increased LC3-II/LC3-I ratio	MPC5 and MSC1097 moues podocytes MPC5 and mesangial cells MSC1097 (Nephropathy), acute spinal cord injured rat model (neurodegenerative disease)	[166,167]
	mTOR inhibition, increased LC3-II, Atg5, and Atg7, and p62 reduction	Cecal ligation and puncture-induced mice model (septic encephalopathy)	[168]
	mTOR inhibition	Primary rat hippocampal neurons (neurodegenerative disease)	[169]
	Activation of MEK/ERK signaling, upregulation of Noxa	Rat model of aortic banding-induced hypertrophy (cardiac Hypertrophy)	[170]
Resveratrol	cAMP/PRKA/AMPK/SIRT1 activation	Human umbilical vein endothelial cell (endothelial inflammation, atherosclerosis)	[94]
	ATG4 restoration	SH-SY5Y cells hyper-expressing the mutant polyQ Huntingtin protein (Huntington’s Disease)	[171]
	Suppression of microRNA-383-5p	Human podocytes, db/db mice (diabetic nephropathy)	[172]
	Inhibition of mTOR/ULK1 signaling	MCF7 cell (human breast cancer), PC3 and DU145 cells (human prostate cancer)	[173,174]
	STAT3 inhibition	CAOV-3 and OVCAR-3 cells (human ovarian cancer)	[175]
	AMPK activation, mTOR inhibition	Cisplatin-resistant human oral cancer cells, destabilization of the medial meniscus in mice (osteoarthritis cartilage)	[176,177]
Rottlerin	Inhibition of PI3K/Akt/mTOR signaling	Cancer stem cells	[178,179,180]
Saikosaponin-d	Inhibition of SERCA, leading to increase in Ca2^+^ and activation of CaMKKβ	HeLa and MCF-7 (human cervical and breast cancer)	[181]
Salvianolic acid B	Akt/mTOR inhibition	HCT116 and HT29 cell (human colorectal cancer)	[182]
Salvigenin	Increased LC3-II/LC3-I, Atg7, and Atg12 in the presence of H_2_O_2_	SH-SY5Y cell (human neuroblastoma, neurodegenerative disorders)	[183]
Spermidine	Increased titin phosphorylation, suppression of inflammation	Cardiomyocytes, mice, and Dahl salt-sensitive rats fed a High-salt diet (cardiovascular disease)	[80]
	Modulation of SIRT1-indipendent deacetylation pathway	HCT 116 cells, *C. elegans*, and cytoplasts (longevity)	[81]
	Inhibition of the caspase 3/Beclin 1 cleavage	PC12 cells and cortical neurons (neurodegenerative disease)	[184]
	Increased cholesterol efflux	High-fat fed ApoE^−/−^ mice (atherosclerosis)	[185]
Trehalose	mTOR-independent autophagy induction	Human foreskin fibroblasts, human aortic endothelial cells (human cytomegalovirus, atherosclerosis)	[88]
	Impaired IFN-λ1	Human primary airway epithelial cells (human rhinovirus, asthma)	[91]
	mTOR-independent autophagy induction, reduction of mutant huntingtin aggregates	COS-7, SK-N-SH, HeLa, and HeLa cells expressing EGFP-LC3, Atg5-deficient and wild type mouse embryonic fibroblasts, PC12 (huntington’s disease and Parkinson’s disease)	[83]
	Increase in TFEB expression and nuclear localization	ApoE^−/−^ and wild-type mice (atherosclerosis)	[186]
Vitamin D3	Human cathelicidin-dependent activation of Beclin-1 and Atg5	Human monocytes and macrophages (tuberculosis)	[97]
	Increased LC3, and Beclin 1	MIN6 mouse insulinoma β-cell and streptozotocin-induced mice model (type 1 diabetes)	[187]
	Up-regulation of LC3-II/LC3-I and PR-39 mRNA expression	Porcine small intestinal epithelial cell, pig model (rotavirus infection)	[188]
Vorinostat	HDAC inhibition	U937 and SUDHL6 cell (hematological cancer)	[189]

*BNIP3*, Bcl-2/adenovirus E1B 19-kDa-interacting protein 3; *JNK*, c-Jun N-terminal kinase; *CEBPD*, Transcription factor CCAAT/enhancer-binding protein delta; *HSV-1*, Herpes simplex virus type 1; *LC*, microtubule-associated protein 1A/1B-light chain; *SERCA*, sarcoplasmic/endoplasmic reticulum Ca^2+^ ATPase; *CaMKKβ*, Ca^2+^/calmodulin-dependent kinase kinase-β; *HCMV*, Human cytomegalovirus; *TFEB*, transcription factor EB; *HDAC*, Histone deacetylase.

## References

[1] Mathew R., Karantza-Wadsworth V., White E. (2007). Role of autophagy in cancer. Nat. Rev. Cancer.

[2] White E. (2012). Deconvoluting the context-dependent role for autophagy in cancer. Nat. Rev. Cancer.

[3] Clague M.J., Urbe S. (2010). Ubiquitin: Same molecule, different degradation pathways. Cell.

[4] Levine B., Kroemer G. (2008). Autophagy in the pathogenesis of disease. Cell.

[5] Kirkin V., McEwan D.G., Novak I., Dikic I. (2009). A role for ubiquitin in selective autophagy. Mol. Cell.

[6] Levine B., Mizushima N., Virgin H.W. (2011). Autophagy in immunity and inflammation. Nature.

[7] Kovacs J., Li C., Lu B. (2010). Beclin-1 is required for T cell-mediated immune responses. J. Immnol..

[8] Saitoh T., Fujita N., Jang M.H., Uematsu S., Yang B.G., Satoh T., Omori H., Noda T., Yamamoto N., Komatsu M. (2008). Loss of the autophagy protein atg16l1 enhances endotoxin-induced il-1beta production. Nature.

[9] Ma Y., Galluzzi L., Zitvogel L., Kroemer G. (2013). Autophagy and cellular immune responses. Immunity.

[10] Deretic V., Saitoh T., Akira S. (2013). Autophagy in infection, inflammation and immunity. Nat. Rev. Immunol..

[11] Delgado M.A., Elmaoued R.A., Davis A.S., Kyei G., Deretic V. (2008). Toll-like receptors control autophagy. EMBO J..

[12] Lee M.S., Kim Y.J. (2007). Signaling pathways downstream of pattern-recognition receptors and their cross talk. Ann. Rev. Biochem..

[13] Jounai N., Kobiyama K., Shiina M., Ogata K., Ishii K.J., Takeshita F. (2011). NLRP4 negatively regulates autophagic processes through an association with beclin1. J. Immunol..

[14] Castillo E.F., Dekonenko A., Arko-Mensah J., Mandell M.A., Dupont N., Jiang S., Delgado-Vargas M., Timmins G.S., Bhattacharya D., Yang H. (2012). Autophagy protects against active tuberculosis by suppressing bacterial burden and inflammation. Proc. Natl. Acad. Sci. USA.

[15] Watson R.O., Manzanillo P.S., Cox J.S. (2012). Extracellular M. tuberculosis DNA targets bacteria for autophagy by activating the host DNA-sensing pathway. Cell.

[16] Kimmey J.M., Stallings C.L. (2016). Bacterial Pathogens versus Autophagy: Implications for Therapeutic Interventions. Trends Mol. Med..

[17] Kimmey J.M., Huynh J.P., Weiss L.A., Park S., Kambal A., Debnath J., Virgin H.W., Stallings C.L. (2015). Unique role for ATG5 in neutrophil-mediated immunopathology during M. tuberculosis infection. Nature.

[18] Martins J.D., Liberal J., Silva A., Ferreira I., Neves B.M., Cruz M.T. (2015). Autophagy and inflammasome interplay. DNA Cell Biol..

[19] Bronietzki A.W., Schuster M., Schmitz I. (2015). Autophagy in T-cell development, activation and differentiation. Immunol. Cell Biol..

[20] Dengjel J., Schoor O., Fischer R., Reich M., Kraus M., Muller M., Kreymborg K., Altenberend F., Brandenburg J., Kalbacher H. (2005). Autophagy promotes MHC class II presentation of peptides from intracellular source proteins. Proc. Natl. Acad. Sci. USA.

[21] Munz C. (2012). Antigen Processing for MHC Class II Presentation via Autophagy. Front. Immunol..

[22] Zhou D., Li P., Lin Y., Lott J.M., Hislop A.D., Canaday D.H., Brutkiewicz R.R., Blum J.S. (2005). Lamp-2a facilitates MHC class II presentation of cytoplasmic antigens. Immunity.

[23] Crotzer V.L., Blum J.S. (2009). Autophagy and its role in MHC-mediated antigen presentation. J. Immunol..

[24] Botbol Y., Guerrero-Ros I., Macian F. (2016). Key roles of autophagy in regulating T-cell function. Eur. J. Immunol..

[25] Pua H.H., Guo J., Komatsu M., He Y.-W. (2009). Autophagy is essential for mitochondrial clearance in mature T lymphocytes. J. Immunol..

[26] Watanabe R., Fujii H., Shirai T., Saito S., Ishii T., Harigae H. (2014). Autophagy plays a protective role as an anti-oxidant system in human T cells and represents a novel strategy for induction of T-cell apoptosis. Eur. J. Immunol..

[27] Jia W., Pua H.H., Li Q.J., He Y.W. (2011). Autophagy regulates endoplasmic reticulum homeostasis and calcium mobilization in T lymphocytes. J. Immunol..

[28] Stephenson L.M., Miller B.C., Ng A., Eisenberg J., Zhao Z., Cadwell K., Graham D.B., Mizushima N.N., Xavier R., Virgin H.W. (2009). Identification of Atg5-dependent transcriptional changes and increases in mitochondrial mass in Atg5-deficient T lymphocytes. Autophagy.

[29] Kovacs J., Li C., Yang Q., Li G., Garcia I., Ju S., Roodman D., Windle J., Zhang X., Lu B. (2012). Autophagy promotes T-cell survival through degradation of proteins of the cell death machinery. Cell Death Differ..

[30] Botbol Y., Patel B., Macian F. (2015). Common gamma-chain cytokine signaling is required for macroautophagy induction during CD4+ T-cell activation. Autophagy.

[31] Hubbard V.M., Valdor R., Patel B., Singh R., Cuervo A.M., Macian F. (2010). Macroautophagy regulates energy metabolism during effector T cell activation. J. Immunol..

[32] Paul S., Kashyap A.K., Jia W., He Y.W., Schaefer B.C. (2012). Selective autophagy of the adaptor protein Bcl10 modulates T cell receptor activation of NF-kappaB. Immunity.

[33] Xu X., Araki K., Li S., Han J.H., Ye L., Tan W.G., Konieczny B.T., Bruinsma M.W., Martinez J., Pearce E.L. (2014). Autophagy is essential for effector CD8(+) T cell survival and memory formation. Nat. Immunol..

[34] Kingeter L.M., Paul S., Maynard S.K., Cartwright N.G., Schaefer B.C. (2010). Cutting edge: TCR ligation triggers digital activation of NF-kappaB. J. Immunol..

[35] Puleston D.J., Zhang H., Powell T.J., Lipina E., Sims S., Panse I., Watson A.S., Cerundolo V., Townsend A.R., Klenerman P. (2014). Autophagy is a critical regulator of memory CD8(+) T cell formation. ELife.

[36] Zhou X.J., Lu X.L., Lv J.C., Yang H.Z., Qin L.X., Zhao M.H., Su Y., Li Z.G., Zhang H. (2011). Genetic association of PRDM1-ATG5 intergenic region and autophagy with systemic lupus erythematosus in a Chinese population. Ann. Rheum. Dis..

[37] Lin N.Y., Beyer C., Giessl A., Kireva T., Scholtysek C., Uderhardt S., Munoz L.E., Dees C., Distler A., Wirtz S. (2013). Autophagy regulates TNFalpha-mediated joint destruction in experimental arthritis. Ann. Rheum. Dis..

[38] Barrett J.C., Hansoul S., Nicolae D.L., Cho J.H., Duerr R.H., Rioux J.D., Brant S.R., Silverberg M.S., Taylor K.D., Barmada M.M. (2008). Genome-wide association defines more than 30 distinct susceptibility loci for crohn’s disease. Nat. Genet..

[39] Klionsky D.J. (2009). Crohn’s disease, autophagy, and the Paneth cell. N. Engl. J. Med..

[40] Travassos L.H., Carneiro L.A., Ramjeet M., Hussey S., Kim Y.G., Magalhaes J.G., Yuan L., Soares F., Chea E., Le Bourhis L. (2010). Nod1 and Nod2 direct autophagy by recruiting ATG16L1 to the plasma membrane at the site of bacterial entry. Nat. Immunol..

[41] Jin Y., Hong Y., Park C.Y., Hong Y. (2017). Molecular Interactions of Autophagy with the Immune System and Cancer. Int. J. Mol. Sci..

[42] Schreiber R.D., Old L.J., Smyth M.J. (2011). Cancer immunoediting: Integrating immunity’s roles in cancer suppression and promotion. Science.

[43] Couzin-Frankel J. (2013). Breakthrough of the year 2013. Cancer immunotherapy. Science.

[44] Michaud M., Martins I., Sukkurwala A.Q., Adjemian S., Ma Y., Pellegatti P., Shen S., Kepp O., Scoazec M., Mignot G. (2011). Autophagy-dependent anticancer immune responses induced by chemotherapeutic agents in mice. Science.

[45] White E. (2015). The role for autophagy in cancer. J. Clin. Investig..

[46] Yang Z.J., Chee C.E., Huang S., Sinicrope F.A. (2011). The role of autophagy in cancer: Therapeutic implications. Mol. Cancer Ther..

[47] Hosokawa N., Hara T., Kaizuka T., Kishi C., Takamura A., Miura Y., Iemura S., Natsume T., Takehana K., Yamada N. (2009). Nutrient-dependent mTORC1 association with the ULK1-Atg13-FIP200 complex required for autophagy. Mol. Biol. Cell.

[48] Ganley I.G., Lam du H., Wang J., Ding X., Chen S., Jiang X. (2009). ULK1.ATG13.FIP200 complex mediates mTOR signaling and is essential for autophagy. J. Biol. Chem..

[49] Kim J., Kundu M., Viollet B., Guan K.L. (2011). AMPK and mTOR regulate autophagy through direct phosphorylation of Ulk1. Nat. Cell Biol..

[50] Sun K., Deng W., Zhang S., Cai N., Jiao S., Song J., Wei L. (2013). Paradoxical roles of autophagy in different stages of tumorigenesis: Protector for normal or cancer cells. Cell Biosci..

[51] Rao S., Tortola L., Perlot T., Wirnsberger G., Novatchkova M., Nitsch R., Sykacek P., Frank L., Schramek D., Komnenovic V. (2014). A dual role for autophagy in a murine model of lung cancer. Nat. Commun..

[52] Han Q., Deng Y., Chen S., Chen R., Yang M., Zhang Z., Sun X., Wang W., He Y., Wang F. (2017). Downregulation of atg5-dependent macroautophagy by chaperone-mediated autophagy promotes breast cancer cell metastasis. Sci. Rep..

[53] Rohatgi R.A., Janusis J., Leonard D., Bellve K.D., Fogarty K.E., Baehrecke E.H., Corvera S., Shaw L.M. (2015). Beclin 1 regulates growth factor receptor signaling in breast cancer. Oncogene.

[54] Takamura A., Komatsu M., Hara T., Sakamoto A., Kishi C., Waguri S., Eishi Y., Hino O., Tanaka K., Mizushima N. (2011). Autophagy-deficient mice develop multiple liver tumors. Genes Dev..

[55] Corcelle E.A., Puustinen P., Jaattela M. (2009). Apoptosis and autophagy: Targeting autophagy signalling in cancer cells -‘trick or treats’?. FEBS J..

[56] Qu X., Yu J., Bhagat G., Furuya N., Hibshoosh H., Troxel A., Rosen J., Eskelinen E.L., Mizushima N., Ohsumi Y. (2003). Promotion of tumorigenesis by heterozygous disruption of the beclin 1 autophagy gene. J. Clin. Investig..

[57] Liang X.H., Jackson S., Seaman M., Brown K., Kempkes B., Hibshoosh H., Levine B. (1999). Induction of autophagy and inhibition of tumorigenesis by beclin 1. Nature.

[58] Chen Z., Li Y., Zhang C., Yi H., Wu C., Wang J., Liu Y., Tan J., Wen J. (2013). Downregulation of Beclin 1 and impairment of autophagy in a small population of colorectal cancer. Dig. Dis. Sci..

[59] Yue Z., Jin S., Yang C., Levine A.J., Heintz N. (2003). Beclin 1, an autophagy gene essential for early embryonic development, is a haploinsufficient tumor suppressor. Proc. Natl. Acad. Sci. USA.

[60] Degenhardt K., Mathew R., Beaudoin B., Bray K., Anderson D., Chen G., Mukherjee C., Shi Y., Gelinas C., Fan Y. (2006). Autophagy promotes tumor cell survival and restricts necrosis, inflammation, and tumorigenesis. Cancer Cell.

[61] Tan Q., Wang M., Yu M., Zhang J., Bristow R.G., Hill R.P., Tannock I.F. (2016). Role of Autophagy as a Survival Mechanism for Hypoxic Cells in Tumors. Neoplasia.

[62] Marino G., Niso-Santano M., Baehrecke E.H., Kroemer G. (2014). Self-consumption: The interplay of autophagy and apoptosis. Nat. Rev. Mol. Cell Biol..

[63] Jin S., DiPaola R.S., Mathew R., White E. (2007). Metabolic catastrophe as a means to cancer cell death. J. Cell Sci..

[64] White E. (2008). Autophagic cell death unraveled: Pharmacological inhibition of apoptosis and autophagy enables necrosis. Autophagy.

[65] Amaravadi R.K., Yu D., Lum J.J., Bui T., Christophorou M.A., Evan G.I., Thomas-Tikhonenko A., Thompson C.B. (2007). Autophagy inhibition enhances therapy-induced apoptosis in a Myc-induced model of lymphoma. J. Clin. Investig..

[66] Choi K.S. (2012). Autophagy and cancer. Exp. Mol. Med..

[67] Schoenlein P.V., Periyasamy-Thandavan S., Samaddar J.S., Jackson W.H., Barrett J.T. (2009). Autophagy facilitates the progression of ERalpha-positive breast cancer cells to antiestrogen resistance. Autophagy.

[68] Fels D.R., Ye J., Segan A.T., Kridel S.J., Spiotto M., Olson M., Koong A.C., Koumenis C. (2008). Preferential cytotoxicity of bortezomib toward hypoxic tumor cells via overactivation of endoplasmic reticulum stress pathways. Cancer Res..

[69] Claerhout S., Verschooten L., Van Kelst S., De Vos R., Proby C., Agostinis P., Garmyn M. (2010). Concomitant inhibition of AKT and autophagy is required for efficient cisplatin-induced apoptosis of metastatic skin carcinoma. Int. J. Cancer.

[70] Levy J., Cacheux W., Bara M.A., L’Hermitte A., Lepage P., Fraudeau M., Trentesaux C., Lemarchand J., Durand A., Crain A.M. (2015). Intestinal inhibition of atg7 prevents tumour initiation through a microbiome-influenced immune response and suppresses tumour growth. Nat. Cell Biol..

[71] Mathew R., Karp C.M., Beaudoin B., Vuong N., Chen G., Chen H.Y., Bray K., Reddy A., Bhanot G., Gelinas C. (2009). Autophagy suppresses tumorigenesis through elimination of p62. Cell.

[72] Choubey V., Cagalinec M., Liiv J., Safiulina D., Hickey M.A., Kuum M., Liiv M., Anwar T., Eskelinen E.L., Kaasik A. (2014). BECN1 is involved in the initiation of mitophagy: It facilitates PARK2 translocation to mitochondria. Autophagy.

[73] Komatsu M., Waguri S., Koike M., Sou Y.S., Ueno T., Hara T., Mizushima N., Iwata J., Ezaki J., Murata S. (2007). Homeostatic levels of p62 control cytoplasmic inclusion body formation in autophagy-deficient mice. Cell.

[74] Komatsu M., Kurokawa H., Waguri S., Taguchi K., Kobayashi A., Ichimura Y., Sou Y.S., Ueno I., Sakamoto A., Tong K.I. (2010). The selective autophagy substrate p62 activates the stress responsive transcription factor nrf2 through inactivation of keap1. Nat. Cell. Biol..

[75] Harris J. (2013). Autophagy and IL-1 Family Cytokines. Front. Immunol..

[76] Martinez-Outschoorn U.E., Whitaker-Menezes D., Lin Z., Flomenberg N., Howell A., Pestell R.G., Lisanti M.P., Sotgia F. (2011). Cytokine production and inflammation drive autophagy in the tumor microenvironment: Role of stromal caveolin-1 as a key regulator. Cell Cycle.

[77] Kuo S.Y., Castoreno A.B., Aldrich L.N., Lassen K.G., Goel G., Dancik V., Kuballa P., Latorre I., Conway K.L., Sarkar S. (2015). Small-molecule enhancers of autophagy modulate cellular disease phenotypes suggested by human genetics. Proc. Natl. Acad. Sci. USA.

[78] Atiya Ali M., Poortvliet E., Stromberg R., Yngve A. (2011). Polyamines in foods: Development of a food database. Food Nutr. Res..

[79] Eisenberg T., Knauer H., Schauer A., Buttner S., Ruckenstuhl C., Carmona-Gutierrez D., Ring J., Schroeder S., Magnes C., Antonacci L. (2009). Induction of autophagy by spermidine promotes longevity. Nat. Cell Biol..

[80] Eisenberg T., Abdellatif M., Schroeder S., Primessnig U., Stekovic S., Pendl T., Harger A., Schipke J., Zimmermann A., Schmidt A. (2016). Cardioprotection and lifespan extension by the natural polyamine spermidine. Nat. Med..

[81] Morselli E., Marino G., Bennetzen M.V., Eisenberg T., Megalou E., Schroeder S., Cabrera S., Benit P., Rustin P., Criollo A. (2011). Spermidine and resveratrol induce autophagy by distinct pathways converging on the acetylproteome. J. Cell Biol..

[82] Araki K., Turner A.P., Shaffer V.O., Gangappa S., Keller S.A., Bachmann M.F., Larsen C.P., Ahmed R. (2009). Mtor regulates memory cd8 t-cell differentiation. Nature.

[83] Sarkar S., Davies J.E., Huang Z., Tunnacliffe A., Rubinsztein D.C. (2007). Trehalose, a novel mtor-independent autophagy enhancer, accelerates the clearance of mutant huntingtin and alpha-synuclein. J. Biol. Chem..

[84] Mocarski E.S. (2002). Immunomodulation by cytomegaloviruses: Manipulative strategies beyond evasion. Trends Microbiol..

[85] Berencsi K., Endresz V., Klurfeld D., Kari L., Kritchevsky D., Gonczol E. (1998). Early atherosclerotic plaques in the aorta following cytomegalovirus infection of mice. Cell Adhes. Commun..

[86] Rosenfeld M.E., Campbell L.A. (2011). Pathogens and atherosclerosis: Update on the potential contribution of multiple infectious organisms to the pathogenesis of atherosclerosis. Thromb. Haemost..

[87] Cobbs C.S. (2013). Cytomegalovirus and brain tumor: Epidemiology, biology and therapeutic aspects. Curr. Opin. Oncol..

[88] Belzile J.P., Sabalza M., Craig M., Clark E., Morello C.S., Spector D.H. (2015). Trehalose, an mtor-independent inducer of autophagy, inhibits human cytomegalovirus infection in multiple cell types. J. Virol..

[89] Hewitt R., Farne H., Ritchie A., Luke E., Johnston S.L., Mallia P. (2016). The role of viral infections in exacerbations of chronic obstructive pulmonary disease and asthma. Ther. Adv. Respir. Dis..

[90] Stock I. (2014). Human rhinovirus diseases--epidemiology, treatment and prevention. Med. Monatsschr. Pharm..

[91] Wu Q., Jiang D., Huang C., van Dyk L.F., Li L., Chu H.W. (2015). Trehalose-mediated autophagy impairs the anti-viral function of human primary airway epithelial cells. PLoS ONE.

[92] Jackson W.T. (2015). Viruses and the autophagy pathway. Virology.

[93] Duan W.J., Liu F.L., He R.R., Yuan W.L., Li Y.F., Tsoi B., Su W.W., Yao X.S., Kurihara H. (2013). Autophagy is involved in the effects of resveratrol on prevention of splenocyte apoptosis caused by oxidative stress in restrained mice. Mol. Nutr. Food Res..

[94] Chen M.L., Yi L., Jin X., Liang X.Y., Zhou Y., Zhang T., Xie Q., Zhou X., Chang H., Fu Y.J. (2013). Resveratrol attenuates vascular endothelial inflammation by inducing autophagy through the camp signaling pathway. Autophagy.

[95] Hoyer-Hansen M., Nordbrandt S.P., Jaattela M. (2010). Autophagy as a basis for the health-promoting effects of vitamin D. Trends Mol. Med..

[96] Smith I. (2003). Mycobacterium tuberculosis pathogenesis and molecular determinants of virulence. Clin. Microbiol. Rev..

[97] Yuk J.M., Shin D.M., Lee H.M., Yang C.S., Jin H.S., Kim K.K., Lee Z.W., Lee S.H., Kim J.M., Jo E.K. (2009). Vitamin d3 induces autophagy in human monocytes/macrophages via cathelicidin. Cell Host. Microbe.

[98] Wang H., Li D., Li X., Ou X., Liu S., Zhang Y., Ding J., Xie B. (2016). Mammalian target of rapamycin inhibitor rad001 sensitizes endometrial cancer cells to paclitaxel-induced apoptosis via the induction of autophagy. Oncol. Lett..

[99] Takeuchi H., Kondo Y., Fujiwara K., Kanzawa T., Aoki H., Mills G.B., Kondo S. (2005). Synergistic augmentation of rapamycin-induced autophagy in malignant glioma cells by phosphatidylinositol 3-kinase/protein kinase b inhibitors. Cancer Res..

[100] Laplante M., Sabatini D.M. (2012). Mtor signaling in growth control and disease. Cell.

[101] Lui A., New J., Ogony J., Thomas S., Lewis-Wambi J. (2016). Everolimus downregulates estrogen receptor and induces autophagy in aromatase inhibitor-resistant breast cancer cells. BMC Cancer.

[102] Kwitkowski V.E., Prowell T.M., Ibrahim A., Farrell A.T., Justice R., Mitchell S.S., Sridhara R., Pazdur R. (2010). Fda approval summary: Temsirolimus as treatment for advanced renal cell carcinoma. Oncologist.

[103] Hess G., Herbrecht R., Romaguera J., Verhoef G., Crump M., Gisselbrecht C., Laurell A., Offner F., Strahs A., Berkenblit A. (2009). Phase iii study to evaluate temsirolimus compared with investigator’s choice therapy for the treatment of relapsed or refractory mantle cell lymphoma. J. Clin. Oncol..

[104] Markman B., Dienstmann R., Tabernero J. (2010). Targeting the pi3k/akt/mtor pathway—Beyond rapalogs. Oncotarget.

[105] Margolin K., Longmate J., Baratta T., Synold T., Christensen S., Weber J., Gajewski T., Quirt I., Doroshow J.H. (2005). Cci-779 in metastatic melanoma: A phase II trial of the california cancer consortium. Cancer.

[106] Galanis E., Buckner J.C., Maurer M.J., Kreisberg J.I., Ballman K., Boni J., Peralba J.M., Jenkins R.B., Dakhil S.R., Morton R.F. (2005). Phase ii trial of temsirolimus (cci-779) in recurrent glioblastoma multiforme: A north central cancer treatment group study. J. Clin. Oncol..

[107] Chan S., Scheulen M.E., Johnston S., Mross K., Cardoso F., Dittrich C., Eiermann W., Hess D., Morant R., Semiglazov V. (2005). Phase ii study of temsirolimus (cci-779), a novel inhibitor of mtor, in heavily pretreated patients with locally advanced or metastatic breast cancer. J. Clin. Oncol..

[108] Bailey C.J., Day C. (1989). Traditional plant medicines as treatments for diabetes. Diabetes Care.

[109] Pernicova I., Korbonits M. (2014). Metformin--mode of action and clinical implications for diabetes and cancer. Nat. Rev. Endocrinol..

[110] Tomic T., Botton T., Cerezo M., Robert G., Luciano F., Puissant A., Gounon P., Allegra M., Bertolotto C., Bereder J.M. (2011). Metformin inhibits melanoma development through autophagy and apoptosis mechanisms. Cell Death Dis..

[111] Takahashi A., Kimura F., Yamanaka A., Takebayashi A., Kita N., Takahashi K., Murakami T. (2014). Metformin impairs growth of endometrial cancer cells via cell cycle arrest and concomitant autophagy and apoptosis. Cancer Cell Int..

[112] Nazim U.M., Moon J.H., Lee J.H., Lee Y.J., Seol J.W., Eo S.K., Lee J.H., Park S.Y. (2016). Activation of autophagy flux by metformin downregulates cellular flice-like inhibitory protein and enhances trail- induced apoptosis. Oncotarget.

[113] Cerezo M., Tichet M., Abbe P., Ohanna M., Lehraiki A., Rouaud F., Allegra M., Giacchero D., Bahadoran P., Bertolotto C. (2013). Metformin blocks melanoma invasion and metastasis development in ampk/p53-dependent manner. Mol. Cancer Ther..

[114] Viollet B., Guigas B., Sanz Garcia N., Leclerc J., Foretz M., Andreelli F. (2012). Cellular and molecular mechanisms of metformin: An overview. Clin. Sci..

[115] Hur K.Y., Lee M.S. (2015). New mechanisms of metformin action: Focusing on mitochondria and the gut. J. Diabetes Investig..

[116] O’Brien S.M., Claxton D.F., Crump M., Faderl S., Kipps T., Keating M.J., Viallet J., Cheson B.D. (2009). Phase i study of obatoclax mesylate (gx15–070), a small molecule pan-bcl-2 family antagonist, in patients with advanced chronic lymphocytic leukemia. Blood.

[117] Schimmer A.D., O’Brien S., Kantarjian H., Brandwein J., Cheson B.D., Minden M.D., Yee K., Ravandi F., Giles F., Schuh A. (2008). A phase I study of the pan bcl-2 family inhibitor obatoclax mesylate in patients with advanced hematologic malignancies. Clin. Cancer Res..

[118] Bonapace L., Bornhauser B.C., Schmitz M., Cario G., Ziegler U., Niggli F.K., Schafer B.W., Schrappe M., Stanulla M., Bourquin J.P. (2010). Induction of autophagy-dependent necroptosis is required for childhood acute lymphoblastic leukemia cells to overcome glucocorticoid resistance. J. Clin. Investig..

[119] Basit F., Cristofanon S., Fulda S. (2013). Obatoclax (gx15-070) triggers necroptosis by promoting the assembly of the necrosome on autophagosomal membranes. Cell Death Differ..

[120] Martin A.P., Park M.A., Mitchell C., Walker T., Rahmani M., Thorburn A., Haussinger D., Reinehr R., Grant S., Dent P. (2009). Bcl-2 family inhibitors enhance histone deacetylase inhibitor and sorafenib lethality via autophagy and overcome blockade of the extrinsic pathway to facilitate killing. Mol. Pharmacol..

[121] Heidari N., Hicks M.A., Harada H. (2010). Gx15-070 (obatoclax) overcomes glucocorticoid resistance in acute lymphoblastic leukemia through induction of apoptosis and autophagy. Cell Death Dis..

[122] Brem E.A., Thudium K., Khubchandani S., Tsai P.C., Olejniczak S.H., Bhat S., Riaz W., Gu J., Iqbal A., Campagna R. (2011). Distinct cellular and therapeutic effects of obatoclax in rituximab-sensitive and -resistant lymphomas. Br. J. Haematol..

[123] Law B.Y., Chan W.K., Xu S.W., Wang J.R., Bai L.P., Liu L., Wong V.K. (2014). Natural small-molecule enhancers of autophagy induce autophagic cell death in apoptosis-defective cells. Sci. Rep..

[124] Cloonan S.M., Drozgowska A., Fayne D., Williams D.C. (2010). The antidepressants maprotiline and fluoxetine have potent selective antiproliferative effects against burkitt lymphoma independently of the norepinephrine and serotonin transporters. Leuk. Lymphoma.

[125] Reddy K.K., Lefkove B., Chen L.B., Govindarajan B., Carracedo A., Velasco G., Carrillo C.O., Bhandarkar S.S., Owens M.J., Mechta-Grigoriou F. (2008). The antidepressant sertraline downregulates akt and has activity against melanoma cells. Pigment Cell Melanoma Res..

[126] Karlsson H., Gu Y., DePierre J., Nassberger L. (1998). Induction of apoptosis in proliferating lymphocytes by tricyclic antidepressants. Apoptosis.

[127] Stepulak A., Rzeski W., Sifringer M., Brocke K., Gratopp A., Kupisz K., Turski L., Ikonomidou C. (2008). Fluoxetine inhibits the extracellular signal regulated kinase pathway and suppresses growth of cancer cells. Cancer Biol. Ther..

[128] Cloonan S.M., Williams D.C. (2011). The antidepressants maprotiline and fluoxetine induce type ii autophagic cell death in drug-resistant burkitt’s lymphoma. Int. J. Cancer.

[129] Tong X., Smith K.A., Pelling J.C. (2012). Apigenin, a chemopreventive bioflavonoid, induces amp-activated protein kinase activation in human keratinocytes. Mol. Carcinog..

[130] Wang Q., Zeng P., Liu Y., Wen G., Fu X., Sun X. (2015). Inhibition of autophagy ameliorates atherogenic inflammation by augmenting apigenin-induced macrophage apoptosis. Int. Immunopharmacol..

[131] Zhang L., Cheng X., Gao Y., Zheng J., Xu Q., Sun Y., Guan H., Yu H., Sun Z. (2015). Apigenin induces autophagic cell death in human papillary thyroid carcinoma bcpap cells. Food Funct..

[132] Bridgeman B.B., Wang P., Ye B., Pelling J.C., Volpert O.V., Tong X. (2016). Inhibition of mtor by apigenin in uvb-irradiated keratinocytes: A new implication of skin cancer prevention. Cell. Signal..

[133] Kanzawa T., Zhang L., Xiao L., Germano I.M., Kondo Y., Kondo S. (2005). Arsenic trioxide induces autophagic cell death in malignant glioma cells by upregulation of mitochondrial cell death protein bnip3. Oncogene.

[134] Aoki H., Takada Y., Kondo S., Sawaya R., Aggarwal B.B., Kondo Y. (2007). Evidence that curcumin suppresses the growth of malignant gliomas in vitro and in vivo through induction of autophagy: Role of akt and extracellular signal-regulated kinase signaling pathways. Mol. Pharmacol..

[135] Chen D., Tao X., Wang Y., Tian F., Wei Y., Chen G., Shen H., Wang Z., Yu Z., Li H. (2015). Curcumin accelerates reendothelialization and ameliorates intimal hyperplasia in balloon-injured rat carotid artery via the upregulation of endothelial cell autophagy. Int. J. Mol. Med..

[136] Zhang Y., Chen P., Hong H., Wang L., Zhou Y., Lang Y. (2017). Jnk pathway mediates curcumin-induced apoptosis and autophagy in osteosarcoma mg63 cells. Exp. Ther. Med..

[137] Veeran S., Shu B., Cui G., Fu S., Zhong G. (2017). Curcumin induces autophagic cell death in spodoptera frugiperda cells. Pestic. Biochem. Physiol..

[138] Zhao G., Han X., Zheng S., Li Z., Sha Y., Ni J., Sun Z., Qiao S., Song Z. (2016). Curcumin induces autophagy, inhibits proliferation and invasion by downregulating akt/mtor signaling pathway in human melanoma cells. Oncol. Rep..

[139] Yang C., Ma X., Wang Z., Zeng X., Hu Z., Ye Z., Shen G. (2017). Curcumin induces apoptosis and protective autophagy in castration-resistant prostate cancer cells through iron chelation. Drug Des. Dev. Ther..

[140] Tsuyuki S., Fukui S., Watanabe A., Akune S., Tanabe M., Yoshida K. (2012). Delphinidin induces autolysosome as well as autophagosome formation and delphinidin-induced autophagy exerts a cell protective role. J. Biochem. Mol. Toxicol..

[141] Klimaszewska-Wisniewska A., Halas-Wisniewska M., Tadrowski T., Gagat M., Grzanka D., Grzanka A. (2016). Paclitaxel and the dietary flavonoid fisetin: A synergistic combination that induces mitotic catastrophe and autophagic cell death in a549 non-small cell lung cancer cells. Cancer Cell Int..

[142] Suh Y., Afaq F., Khan N., Johnson J.J., Khusro F.H., Mukhtar H. (2010). Fisetin induces autophagic cell death through suppression of mtor signaling pathway in prostate cancer cells. Carcinogenesis.

[143] Zhang X.J., Jia S.S. (2016). Fisetin inhibits laryngeal carcinoma through regulation of akt/nf-kappab/mtor and erk1/2 signaling pathways. Biomed. Pharmacother..

[144] Wang H.X., Tang C. (2017). Galangin suppresses human laryngeal carcinoma via modulation of caspase-3 and akt signaling pathways. Oncol. Rep..

[145] Lee K.Y., Kim J.R., Choi H.C. (2016). Genistein-induced lkb1-ampk activation inhibits senescence of vsmc through autophagy induction. Vascul. Pharmacol..

[146] Benvenuto M., Mattera R., Masuelli L., Taffera G., Andracchio O., Tresoldi I., Lido P., Giganti M.G., Godos J., Modesti A. (2017). (+/−)-gossypol induces apoptosis and autophagy in head and neck carcinoma cell lines and inhibits the growth of transplanted salivary gland cancer cells in balb/c mice. Int. J. Food Sci. Nutr..

[147] Ueda J.Y., Athikomkulchai S., Miyatake R., Saiki I., Esumi H., Awale S. (2014). (+)-grandifloracin, an antiausterity agent, induces autophagic panc-1 pancreatic cancer cell death. Drug Des. Devel. Ther..

[148] Wu M., Lao Y., Xu N., Wang X., Tan H., Fu W., Lin Z., Xu H. (2015). Guttiferone k induces autophagy and sensitizes cancer cells to nutrient stress-induced cell death. Phytomedicine.

[149] Filomeni G., Graziani I., De Zio D., Dini L., Centonze D., Rotilio G., Ciriolo M.R. (2012). Neuroprotection of kaempferol by autophagy in models of rotenone-mediated acute toxicity: Possible implications for parkinson’s disease. Neurobiol. Aging.

[150] Filomeni G., Desideri E., Cardaci S., Graziani I., Piccirillo S., Rotilio G., Ciriolo M.R. (2010). Carcinoma cells activate amp-activated protein kinase-dependent autophagy as survival response to kaempferol-mediated energetic impairment. Autophagy.

[151] Huang W.W., Tsai S.C., Peng S.F., Lin M.W., Chiang J.H., Chiu Y.J., Fushiya S., Tseng M.T., Yang J.S. (2013). Kaempferol induces autophagy through ampk and akt signaling molecules and causes g2/m arrest via downregulation of cdk1/cyclin b in sk-hep-1 human hepatic cancer cells. Int. J. Oncol..

[152] Varshney R., Gupta S., Roy P. (2017). Cytoprotective effect of kaempferol against palmitic acid-induced pancreatic beta-cell death through modulation of autophagy via ampk/mtor signaling pathway. Mol. Cell Endocrinol..

[153] Li H.B., Yi X., Gao J.M., Ying X.X., Guan H.Q., Li J.C. (2007). Magnolol-induced h460 cells death via autophagy but not apoptosis. Arch. Pharm. Res..

[154] Tsai H.H., Lai H.Y., Chen Y.C., Li C.F., Huang H.S., Liu H.S., Tsai Y.S., Wang J.M. (2017). Metformin promotes apoptosis in hepatocellular carcinoma through the cebpd-induced autophagy pathway. Oncotarget.

[155] Lu M., Su C., Qiao C., Bian Y., Ding J., Hu G. (2016). Metformin prevents dopaminergic neuron death in mptp/p-induced mouse model of parkinson’s disease via autophagy and mitochondrial ros clearance. Int. J. Neuropsychopharmacol..

[156] Yao Z., Xie F., Li M., Liang Z., Xu W., Yang J., Liu C., Li H., Zhou H., Qu L.H. (2017). Oridonin induces autophagy via inhibition of glucose metabolism in p53-mutated colorectal cancer cells. Cell Death Dis..

[157] Pei Y., Chen Z.P., Ju H.Q., Komatsu M., Ji Y.H., Liu G., Guo C.W., Zhang Y.J., Yang C.R., Wang Y.F. (2011). Autophagy is involved in anti-viral activity of pentagalloylglucose (pgg) against herpes simplex virus type 1 infection in vitro. Biochem. Biophys. Res. Commun..

[158] Hu H., Chai Y., Wang L., Zhang J., Lee H.J., Kim S.H., Lu J. (2009). Pentagalloylglucose induces autophagy and caspase-independent programmed deaths in human pc-3 and mouse tramp-c2 prostate cancer cells. Mol. Cancer Ther..

[159] Ko C.P., Lin C.W., Chen M.K., Yang S.F., Chiou H.L., Hsieh M.J. (2015). Pterostilbene induce autophagy on human oral cancer cells through modulation of akt and mitogen-activated protein kinase pathway. Oral. Oncol..

[160] Siedlecka-Kroplewska K., Jozwik A., Boguslawski W., Wozniak M., Zauszkiewicz-Pawlak A., Spodnik J.H., Rychlowski M., Kmiec Z. (2013). Pterostilbene induces accumulation of autophagic vacuoles followed by cell death in hl60 human leukemia cells. J. Physiol. Pharmacol..

[161] Psahoulia F.H., Moumtzi S., Roberts M.L., Sasazuki T., Shirasawa S., Pintzas A. (2007). Quercetin mediates preferential degradation of oncogenic ras and causes autophagy in ha-ras-transformed human colon cells. Carcinogenesis.

[162] Moon J.H., Eo S.K., Lee J.H., Park S.Y. (2015). Quercetin-induced autophagy flux enhances trail-mediated tumor cell death. Oncol. Rep..

[163] El-Horany H.E., El-Latif R.N., ElBatsh M.M., Emam M.N. (2016). Ameliorative effect of quercetin on neurochemical and behavioral deficits in rotenone rat model of Parkinson’s disease: Modulating autophagy (quercetin on experimental Parkinson’s disease). J. Biochem. Mol. Toxicol..

[164] Granato M., Rizzello C., Gilardini Montani M.S., Cuomo L., Vitillo M., Santarelli R., Gonnella R., D’Orazi G., Faggioni A., Cirone M. (2017). Quercetin induces apoptosis and autophagy in primary effusion lymphoma cells by inhibiting pi3k/akt/mtor and stat3 signaling pathways. J. Nutr. Biochem..

[165] Wang C., Hu Z., Zou Y., Xiang M., Jiang Y., Botchway B.O.A., Huo X., Du X., Fang M. (2017). The post-therapeutic effect of rapamycin in mild traumatic brain injured rats ensuing in the upregulation of autophagy and mitophagy. Cell Biol. Int..

[166] Liang S., Jin J., Lin B., Gong J., Li Y., He Q. (2017). Rapamycin induces autophagy and reduces the apoptosis of podocytes under a stimulated condition of immunoglobulin a nephropathy. Kidney Blood Press. Res..

[167] Wang Z.Y., Liu W.G., Muharram A., Wu Z.Y., Lin J.H. (2014). Neuroprotective effects of autophagy induced by rapamycin in rat acute spinal cord injury model. Neuroimmunomodulation.

[168] Liu W., Guo J., Mu J., Tian L., Zhou D. (2016). Rapamycin protects sepsis-induced cognitive impairment in mouse hippocampus by enhancing autophagy. Cell Mol. Neurobiol..

[169] Smith E.D., Prieto G.A., Tong L., Sears-Kraxberger I., Rice J.D., Steward O., Cotman C.W. (2014). Rapamycin and interleukin-1beta impair brain-derived neurotrophic factor-dependent neuron survival by modulating autophagy. J. Biol. Chem..

[170] Gu J., Hu W., Song Z.P., Chen Y.G., Zhang D.D., Wang C.Q. (2016). Rapamycin inhibits cardiac hypertrophy by promoting autophagy via the mek/erk/beclin-1 pathway. Front. Physiol..

[171] Vidoni C., Secomandi E., Castiglioni A., Melone M.A.B., Isidoro C. (2017). Resveratrol protects neuronal-like cells expressing mutant huntingtin from dopamine toxicity by rescuing atg4-mediated autophagosome formation. Neurochem. Int..

[172] Huang S.S., Ding D.F., Chen S., Dong C.L., Ye X.L., Yuan Y.G., Feng Y.M., You N., Xu J.R., Miao H. (2017). Resveratrol protects podocytes against apoptosis via stimulation of autophagy in a mouse model of diabetic nephropathy. Sci. Rep..

[173] Park D., Jeong H., Lee M.N., Koh A., Kwon O., Yang Y.R., Noh J., Suh P.G., Park H., Ryu S.H. (2016). Resveratrol induces autophagy by directly inhibiting mtor through atp competition. Sci. Rep..

[174] Selvaraj S., Sun Y., Sukumaran P., Singh B.B. (2016). Resveratrol activates autophagic cell death in prostate cancer cells via downregulation of stim1 and the mtor pathway. Mol. Carcinog..

[175] Zhong L.X., Zhang Y., Wu M.L., Liu Y.N., Zhang P., Chen X.Y., Kong Q.Y., Liu J., Li H. (2016). Resveratrol and stat inhibitor enhance autophagy in ovarian cancer cells. Cell Death Discov..

[176] Chang C.H., Lee C.Y., Lu C.C., Tsai F.J., Hsu Y.M., Tsao J.W., Juan Y.N., Chiu H.Y., Yang J.S., Wang C.C. (2017). Resveratrol-induced autophagy and apoptosis in cisplatin-resistant human oral cancer car cells: A key role of ampk and akt/mtor signaling. Int. J. Oncol..

[177] Qin N., Wei L., Li W., Yang W., Cai L., Qian Z., Wu S. (2017). Local intra-articular injection of resveratrol delays cartilage degeneration in c57bl/6 mice by inducing autophagy via ampk/mtor pathway. J. Pharmacol. Sci..

[178] Singh B.N., Kumar D., Shankar S., Srivastava R.K. (2012). Rottlerin induces autophagy which leads to apoptotic cell death through inhibition of pi3k/akt/mtor pathway in human pancreatic cancer stem cells. Biochem. Pharmacol..

[179] Kumar D., Shankar S., Srivastava R.K. (2013). Rottlerin-induced autophagy leads to the apoptosis in breast cancer stem cells: Molecular mechanisms. Mol. Cancer.

[180] Kumar D., Shankar S., Srivastava R.K. (2014). Rottlerin induces autophagy and apoptosis in prostate cancer stem cells via pi3k/akt/mtor signaling pathway. Cancer Lett..

[181] Wong V.K., Li T., Law B.Y., Ma E.D., Yip N.C., Michelangeli F., Law C.K., Zhang M.M., Lam K.Y., Chan P.L. (2013). Saikosaponin-d, a novel serca inhibitor, induces autophagic cell death in apoptosis-defective cells. Cell Death Dis..

[182] Jing Z., Fei W., Zhou J., Zhang L., Chen L., Zhang X., Liang X., Xie J., Fang Y., Sui X. (2016). Salvianolic acid b, a novel autophagy inducer, exerts antitumor activity as a single agent in colorectal cancer cells. Oncotarget.

[183] Rafatian G., Khodagholi F., Farimani M.M., Abraki S.B., Gardaneh M. (2012). Increase of autophagy and attenuation of apoptosis by salvigenin promote survival of sh-sy5y cells following treatment with h(2)o(2). Mol. Cell. Biochem..

[184] Yang Y., Chen S., Zhang Y., Lin X., Song Y., Xue Z., Qian H., Wang S., Wan G., Zheng X. (2017). Induction of autophagy by spermidine is neuroprotective via inhibition of caspase 3-mediated beclin 1 cleavage. Cell Death Dis..

[185] Michiels C.F., Kurdi A., Timmermans J.P., De Meyer G.R.Y., Martinet W. (2016). Spermidine reduces lipid accumulation and necrotic core formation in atherosclerotic plaques via induction of autophagy. Atherosclerosis.

[186] Sergin I., Evans T.D., Zhang X., Bhattacharya S., Stokes C.J., Song E., Ali S., Dehestani B., Holloway K.B., Micevych P.S. (2017). Exploiting macrophage autophagy-lysosomal biogenesis as a therapy for atherosclerosis. Nat. Commun..

[187] Wang Y., He D., Ni C., Zhou H., Wu S., Xue Z., Zhou Z. (2016). Vitamin d induces autophagy of pancreatic beta-cells and enhances insulin secretion. Mol. Med. Rep..

[188] Tian G., Liang X., Chen D., Mao X., Yu J., Zheng P., He J., Huang Z., Yu B. (2016). Vitamin d3 supplementation alleviates rotavirus infection in pigs and ipec-j2 cells via regulating the autophagy signaling pathway. J. Steroid Biochem. Mol. Biol..

[189] Dupere-Richer D., Kinal M., Menasche V., Nielsen T.H., Del Rincon S., Pettersson F., Miller W.H. (2013). Vorinostat-induced autophagy switches from a death-promoting to a cytoprotective signal to drive acquired resistance. Cell Death Dis..

